# Meibomian Glands or Not? Identification of In Vivo and Ex Vivo Confocal Microscopy Features and Histological Correlates in the Eyelid Margin

**DOI:** 10.1155/2020/7516286

**Published:** 2020-06-30

**Authors:** Yu-jing Wang, Min Ke

**Affiliations:** Department of Ophthalmology, Zhongnan Hospital of Wuhan University, Wuhan 430060, Hubei, China

## Abstract

**Purpose:**

In vivo confocal laser scanning microscopy (CLSM) is an emerging diagnostic tool allowing fast and easy microscopic tissue examination. For the diagnostics of pathological eyelid margin lesions, the knowledge of the normal eyelid margin is essential.

**Methods:**

We examined 18 eyelid margins of healthy humans using the in vivo CLSM device and 10 samples of healthy eyelid margins from donor sites with ex vivo CLSM and compared the findings to the corresponding histological sections of donor sites. Cross-section images of different depths and depths of different skin appendages were measured.

**Results:**

The depth observed by in vivo CLSM is less than 150 *μ*m into the eyelid. Images of the epidermis and superficial dermis skin, appendages including hair follicle, and sebaceous catheters can be captured associated with histopathology and ex vivo confocal microscopy. In correlation with histopathology, we identified different layers of the eyelid margin, different layers of the epidermis, and skin appendages by ex vivo confocal microscopy.

**Conclusions:**

The study offers an overview of the in vivo confocal microscopy human eyelid margin characteristics in comparison to the standard histological examination and confirms that in vivo CLSM could not observe the meibomian gland acini structure.

## 1. Introduction

Blepharitis, an inflammatory condition of the eyelids, occurs in a high proportion (>35%) of patients [1]. For blepharitis, developing objective microscopic examination methods could facilitate one to characterize affected patients and understand the development of the disease over time, which are key requirements for clinical trials.

The confocal laser scanning microscopy (CLSM) is based on the different refraction indices of different tissue structures, and it can observe the cellular level in vivo through high magnification. In 1990, Cavanagh first performed a human eye biopsy using in vivo CLSM to obtain a layered image of the cornea [2]. In vivo CLSM has been successfully used primarily in the corneal field, especially for keratitis and therapeutic monitoring. It allowed noninvasive optical biopsy to produce fast, repeatable, and painless images, and the instrument has been extended for conjunctival disease, including ocular surface squamous neoplasia [3], primary acquired melanosis [4], allergic conjunctivitis [5], pterygium [6], and Demodex infection [7]. The commonly used in vivo CLSM device in ophthalmology, HRT III/RCM (Heidelberg Retinal Tomograph/Rostock Corneal Module) confocal microscope (Heidelberg Engineering, Dossenheim, Germany) uses a 670 nm near-infrared laser. The laser power limits the penetration depth less than 200 *μ*m [8]. Compared to its in vivo counterpart, ex vivo CLSM gives the opportunity to examine all tissue layers without limitation of the penetration depth in the traditional vertical view that can be easily compared to histology. This is achieved by fixing horizontal slices of the excised tissue in an observation chamber and scanning through the whole sample horizontally.

Over the past decade, there have been a considerable number of clinical studies of in vivo CLSM on eyelid margin diseases, including meibomian gland dysfunction, but based on the depth of confocal observation, we are skeptical whether the meibomian glands can be observed. In addition, to the best of our knowledge, there has been no comparison of healthy human eyelid margin by these three methods until now. The purpose of this study was to describe the anatomy of the healthy eyelid margin, as seen in in vivo and ex vivo CLSM, associated with the corresponding histopathology as a basic knowledge of further microscopic examination of pathological eyelid margin lesions. It provides a theoretical basis for the rapid diagnosis of corresponding eyelid diseases and the monitoring of the treatment process.

## 2. Materials and Methods

This study was approved by the institutional review board of Zhongnan Hospital of Wuhan University, Hubei, China, and the Ethics Committee of the Zhongnan Hospital of Wuhan University, Hubei, China. Prior to enrollment in the study, all patients gave written informed consent. This study adhered to the tenets of the Declaration of Helsinki.

From 2017 to 2018, we examined 18 eyelid margins of healthy humans using the in vivo CLSM (Heidelberg Engineering, Germany, HRT III/RCM) device. Inclusion criteria for this study included any adult without any evidence of existing ocular or systemic disease and no use of any topical ophthalmic medication. And 10 specimens of healthy margins around the melanocytic nevus at the eyelid margin examined by ex vivo CLSM (Heidelberg Engineering, Germany, HRT III/RCM) were taken from the Department of Pathology, Zhongnan Hospital of Wuhan University.

### 2.1. In Vivo CLSM Imaging

In vivo CLSM was performed with the HRT III/RCM using a standard operating procedure by an experienced ophthalmologist trained in performing in vivo CLSM. A sterile protective cap (TomoCap; Heidelberg Engineering) was mounted over the front of the microscope. After the examiner asked the patient to look down, the cotton swab was used to flip the upper eyelid, and the center of the TomoCap was applanated onto the upper eyelid edge vertically. The upper eyelid of all eyes was evaluated with in vivo CLSM. Focal distance was modified to evaluate the whole layer until the picture cannot be clearly imaged.

### 2.2. H&E Staining and Ex Vivo CLSM Imaging

To enable the exact correlation to in vivo CLSM, all samples were sectioned horizontally from the epidermis to the dermis into tissue sections of 5 *μ*m thickness. After standard staining with hematoxylin and eosin (H&E) used for the histological section, image acquisition and the ex vivo CLSM (HRT III/RCM) analysis were performed. Two trained CLSM specialists with histological skills performed independent confocal and histological examination of the specimens and evaluated it.

### 2.3. Statistics

The histopathological epidermis thickness and in vivo and ex vivo CLSM confocal epidermis thickness were measured independently and evaluated using correlation curves, Spearman's correlation coefficient.

## 3. Results

We examined 18 eyelid margins of healthy humans using the in vivo CLSM device (male: female ratio = 8 : 10; age between 21 and 40 years, median 30 year) and 10 samples of healthy eyelid margins (male: female ratio = 4 : 6; age between 34 and 45 years, median 37 year) from donor sites by ex vivo CLSM. We focus on identifying the epidermis and the dermis. In addition, we describe various appendages (CLSM images of eyelashes, hair follicles, sebaceous glands, and sweat glands) and subcutaneous tissue, including adipose tissue, collagen, blood vessels, and meibomian glands.

### 3.1. Overview

Spearman's correlation coefficient of the histopathological epidermis thickness and the in vivo and ex vivo CLSM confocal epidermis thickness was 1.00. The maximum examination depth is limited to about 150 *μ*m and 200 *μ*m from the anterior edge (skin) to the posterior edge (palpebral conjunctiva). The main in vivo and ex vivo CLSM microscopic features and histological correlations are shown in Tables 1 and 2.

### 3.2. Epithelium

**Figure 1:**
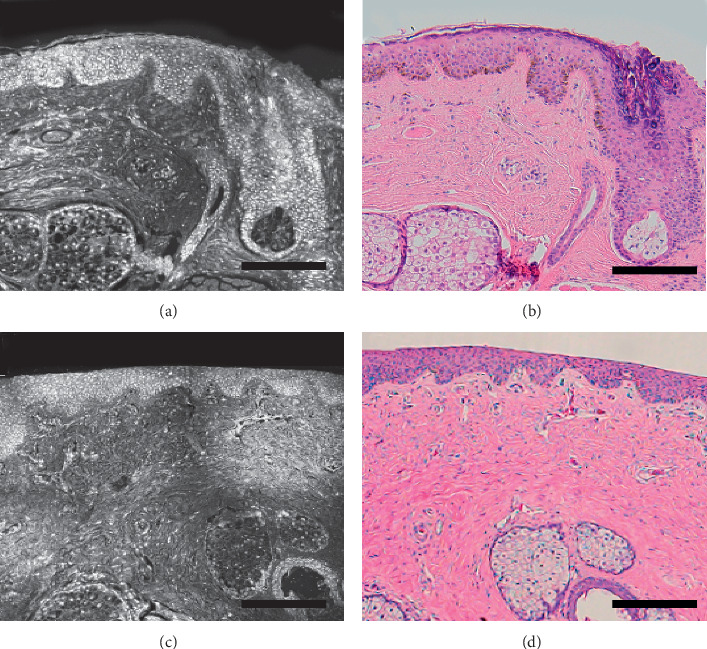
The longitudinal section of the eyelid margin. Keratinized squamous epithelium and skin appendages including hair follicles and sebaceous glands are visible on the skin part: (a) ex vivo confocal microscopy mode; (b) H&E stained histological section. Nonkeratinized squamous epithelium and meibomian glands in the conjunctival part (c) ex vivo confocal microscopy mode; (d) H&E stained histological section (scale bar: 100 *μ*m).

At the edge of the eyelid from the skin to the conjunctival surface, the epidermis is transformed from a keratinized squamous epithelium to a nonkeratinized squamous epithelium. The thickness gradually decreases from about 100 *μ*m to 70 *μ*m. The longitudinal section of the conjunctival surface and that of the skin surface are shown in Figure 1.

### 3.3. Dermoepidermal Junction

**Figure 2:**
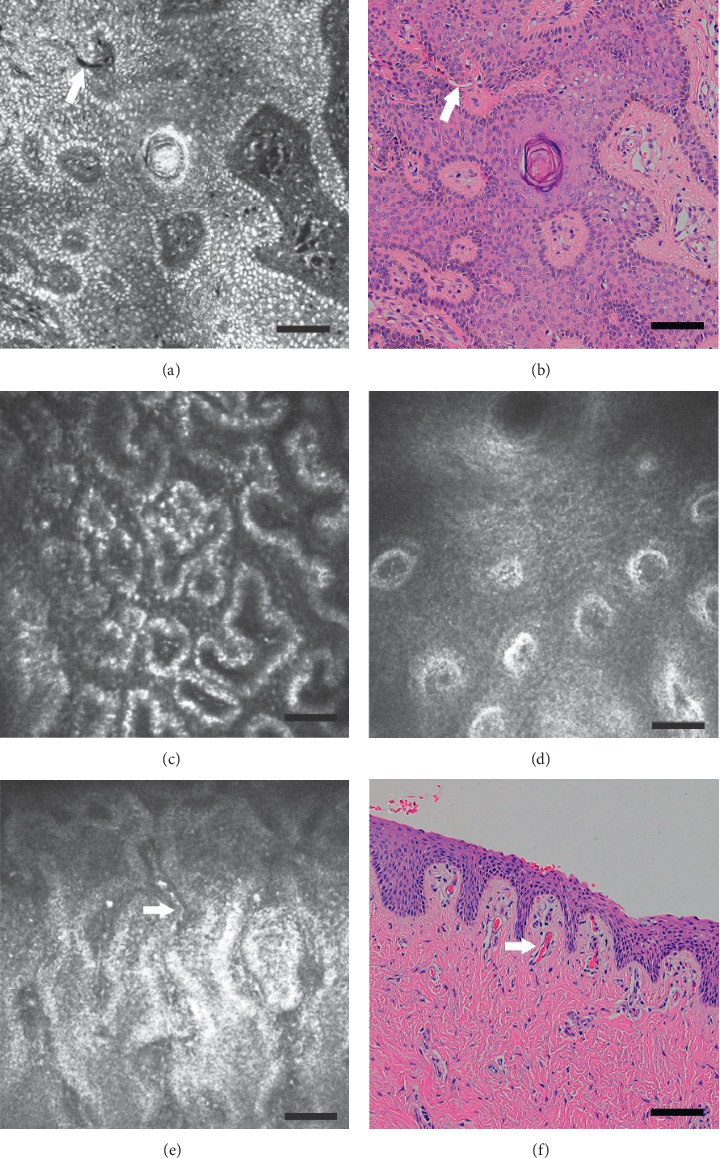
The cross-section of the dermoepidermal junction of the eyelid margin: (a) ex vivo confocal microscopy mode; (b) H&E stained histological section. In this part, the epidermis basement membrane surrounds the dermal papilla. In vivo confocal mode shows that the dermoepidermal junction is composed of a low-reflection area surrounded by a highly reflective layer. The dermal papilla of the skin part has a larger diameter, a higher density, and a polygonal shape (c). The conjunctival part has small diameter, low density, round shape (d). Flowing blood cells are visible in some of the dermal papilla: (e) In vivo confocal microscopy mode; the longitudinal section of H&E stained dermal papilla (scale bar: 50 *μ*m). Arrowheads mark capillaries filled with blood cell.

The dermoepidermal junction consists of the epidermal basal membrane and dermal papillae. As the imaging depth increased from 40 to 100 *μ*m below the epithelial surface, dermal papillae appeared to grow and merge with each other, as shown in Figure 2. Distinct regions across the eyelid margin were visible by in vivo CLSM, and from the skin to the conjunctiva, the size and density of the dermal papilla gradually decrease.

### 3.4. Appendages and Subcutis

**Figure 3:**
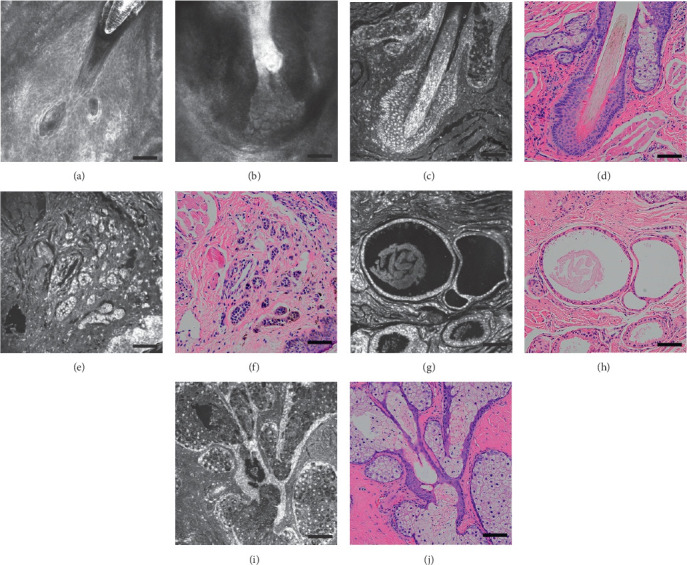
Appendages and subcutis. In vivo confocal microscopy mode can only observe superficial hair follicles (a) and parts of glands of Zeis (b) opens into the hair follicle. The follicles, gland of Zeis ((c) ex vivo confocal microscopy mode; (d) H&E stained section), and eccrine gland ((e) ex vivo confocal microscopy mode; (f) H&E stained section) can be observed on the superficial layer of the skin, the gland of Moll ((g) ex vivo confocal microscopy mode; (h) H&E stained section) can be observed in the deep layer. The meibomian gland ((i) ex vivo confocal microscopy mode; (j) H&E stained section) can be observed in the deep layer of the conjunctiva (scale bar: 50 *μ*m).

The anatomy of skin appendages can be easily viewed and studied by ex vivo CLSM. Only some superficial skin appendages can be seen using in vivo CLSM. Hair and hair follicle performances are similar in both the CLSM methods. Gland of Zeis produced an oily substance that is issued through the excretory ducts of the sebaceous lobule into the middle portion of the hair follicle. It shows lobulation and cellular structure in ex vivo CLSM clearly but only high-reflection catheters and fuzzy medium-reflective lobulated structures in in vivo CLSM. Glands of Moll and meibomian gland are too deep to be observed with in vivo CLSM. The comparison of all skin appendages among the three methods is shown in Figure 3.

## 4. Discussion

In correlation with histopathology, we identified different layers of the eyelid margin and different layers of the epidermis and described in detail skin appendages including hair follicle, sebaceous and sweat glands, conjunctival epithelial cells, and matrix fibers by ex vivo CLSM. Ex vivo CLSM, in contrast to in vivo CLSM, is an invasive diagnostic tool which, owing to that, allows examining the tissue sample in a vertical view. Therefore, the results enable more accurate correlation to histological images. In this experiment, the depth observed by in vivo CLSM is less than 150 *μ*m into the eyelid. Therefore, only the images of the epidermis, superficial dermis skin, and appendages including hair follicle and sebaceous catheters can be captured associated with histopathology. In vivo CLSM has the smallest range among the three detection methods.

The human eyelid margin epidermis is composed of three different regions: skin epidermis, mucocutaneous junction (MCJ), and palpebral conjunctiva [9]. Skin epidermis consists of flat keratinocytes; however, palpebral conjunctiva shows a stratified squamous nonkeratinized epithelium. The MCJ of the human eyelid was first descripted precisely by Knop [10] as characterized by the presence of both parakeratinized cells and discontinuous parakeratinized epithelial cells located in the superficial layer of the epithelium. Considering in vivo and ex vivo confocal microscopy cannot distinguish the mucocutaneous junction. We only describe the skin epidermis and palpebral conjunctiva and the tissue underneath it. We found through in vivo CLSM that palpebral conjunctiva had a clear dividing line at the edge of the eyelid margin. From the skin to the conjunctiva, the superficial epidermal cells are transformed from low-reflecting cells to highly reflective cells with clear boundaries.

The meibomian gland is a type of sebaceous gland with tubuloacinar structure and holocrine function [11]. The hyperreflective, web-like structures observed by in vivo CLSM in the eyelid margin that were presumed to be meibomian glands (MGs) was first published by Kobayashi [12]. Over the past decade, these structures have been studied as MGs, with most studies focusing on changes in reflectance, gland size and density, shape, secretion, and periglandular tissue [13, 14] in diseases such as meibomian gland dysfunction [15, 16], atopic keratoconjunctivitis [17], primary chronic dacryocystitis [18], Sjogren's syndrome [19], and contact lens wearers [20]. In this study, we correlated with histology by in vivo and in vitro confocal microscopy, and described the collection of acinar-like structures with a hyperreflective epithelial layer surrounding a hyporeflective luminal center and distinguished them from the meibomian glands, confirming that the previously studied meibomian glands are actually cross sections of the papillary dermis.

First of all, the site of the meibomian gland and the papillary dermis is different. Previous histological studies have shown that the excretory duct of the MG extends approximately 500 *μ*m beneath the epidermis of the free lid margin before reaching MG acini [11]. However, the papillary dermis is 50 *μ*m beneath the epidermis. Therefore, the current in vivo CLSM wavelength cannot detect the depth of the meibomian glands. Based upon the appearance of human MGs in a cross-sectioned tissue, there are approximately 10–15 acini per MG, with 40 MGs across the entire upper eyelid; the acini are approximately 150–200 *μ*m in diameter clustering around the meibomian gland ducts [21]. In contrast to this, we found that the area of the structures visible in the eyelid margin using in vivo CLSM was less than 100 *μ*m in diameter, smaller than the area of the MG acini. The density is evenly distributed in the horizontal direction, but gradually decreases from the part of the skin rich in eyelashes to the edge of the posterior margin. Second, according to our findings, anatomy of MGs in ex vivo CLSM presents as round, thin, sharply demarcated structures filled with multiple round cells with very thin bright walls, showing bright nuclei and grey cytoplasm filled with large dark cells. Anatomy of papillary dermis in ex vivo CLSM and in vivo CLSM presents as multishaped, multilayered, bright small cuboidal structures filled with reticulated network of fine grey fibers or thicker bundles and fine particles that may be inflammatory cells. In addition, we observed blood flow signals in web-like structures captured by in vivo CLSM, which are abundant in the dermal tissue and unlikely to appear in the glandular tissue. Dermatologists have adequately observed papillary dermis by in vivo CLSM. These studies include those of dermatologic lesions of the human eyelid [22, 23, 24], supporting the argument that the in vivo CLSM structures present in the lid margin are not MG.

As far as we know, this is the first study to focus on in vivo and ex vivo confocal images of the healthy eyelid margin and its appendages. The purpose of this work is to identify and characterize the major characteristics of in vivo confocal of the eyelid margin and to associate them with the traditional ones. Our experiment showed that the in vivo CLSM images of the eyelid margin had a good correlation with the traditional ones, which provided an objective comparison for the in vivo CLSM images collection of the eyelid margin disease. In addition, we systematically summarized the standardized in vivo CLSM images of the healthy eyelid margins. Familiar with in vivo and ex vivo CLSM, the healthy eyelid margin images can understand the pathological processes faster and more accurately.

Ex vivo CLSM has a unique possibility compared to other invasive procedures; the specimen examined with ex vivo CLSM can be reexamined using traditional histology including immunohistochemistry [25]. In this study, the ex vivo CLSM structure of a healthy eyelid is basically the same as the structure of a healthy head, neck, and limbs structure previously studied. However, the epidermis of the eyelid is thinner than the rest, and it has a unique meibomian gland mechanism. Ex vivo CLSM has been used for rapid identification of skin tumor slices, but the diagnosis of the tumor tissue has not been used for eyelid tumors [26]; so, it is a promising research direction. The purpose of this study is to explore the imaging of the eyelid structure of normal people. We will continue this research to provide new ideas for future diagnostic methods in the future.

In vivo CLSM is a new emerging diagnostic tool, which allows fast and easy microscopic tissue examination. In the last decade, the CLSM has developed into a widespread and useful diagnostic method in the field of dermatooncology, inflammatory dermatoses, and therapeutic monitoring [27, 28, 29]. The commonly used in vivo CLSM device in dermatology, VivaScope 1500® (MAVIG GmbH, Munich, Germany), uses an 830 nm near-infrared laser. Morphological details can be defined up to a resolution of 0.5–1.0 *μ*m in the lateral section [30]. The laser power limits the penetration depth to around 250 *μ*m [31]. The histopathological changes of inflammatory skin diseases mainly occur in the epidermis and dermal papilla, and the penetration depth of in vivo CLSM can satisfy the coverage of inflammatory lesions [32]. Dermatologists have assessed the number of dermal papillae, the number of blood vessels in the dermal papillae [33], inflammatory cell density, and inflammatory cell migration [34] by in vivo CLSM to assist in the diagnosis of multiple inflammatory skin diseases. Similarly, we can also use these indicators for the dynamic study of the progression of blepharitis. In addition, in vivo CLSM can also be used to compare drug efficacy and evaluate treatment outcomes. As for the tumor of the eyelid margin, in vivo CLSM might be used to define surgical margins.

Although correlations to classical histological images have been systematically studied and advice for correct handling could be provided, in vivo CLSM is not yet able to replace the traditional histology and immunohistochemistry but might play an important role in future diagnostics implementation. At present, the types of eyelid margin diseases that have been studied are still few, and the sample size needs to be further expanded. There is a lack of uniform imaging diagnostic criteria for marginal lesion and evaluation parameters by CLSM. In addition, in vivo CLSM has limitations in identifying techniques such as accuracy and depth. Therefore, further establishment of imaging diagnostic criteria and determination of the relationship between pathology and imaging are the directions of future research and development. With the continuous development and improvement of technology, in vivo CLSM will better serve the research of ocular surface diseases.

## Figures and Tables

**Table 1:** List of in vivo and ex vivo CLSM features of healthy skin part of the eyelid margin and their histological correlates in H&E staining.

Anatomical	In vivo CLSM	Ex vivo CLSM	H&E staining
Stratum corneum ↓ 0–10 *μ*m	Highly reflective, polygonal, flat, nonnucleated cells (25 *μ*m-35 *μ*m)	Medium reflection, polygonal, flat, nonnucleated cells	Multiple layers of pink, flat, nonnucleated keratinocytes
Stratum granulosum ↓ 10–20 *μ*m	1–3 layers, flat, low-reflective cytoplasm (20–30 *μ*m)	1–3 layers, flat, medium-reflective cytoplasm, highly reflective nuclei	Multiple layers of pink cytoplasm with dark granules and dark blue or purple nuclei
Stratum spinosum ↓ 20–100 *μ*m	2–7 layers, polygon, high-reflective cell junctions, and low-reflective cells with invisible nuclear (15–25 *μ*m)	2–7 layers, polygon, high reflective nucleus, low-reflective cytoplasmic cells	Multilayer, purple nuclei in the center of the pink cytoplasm
Stratum basale ↓ 50–100 *μ*m	Monolayer, cube-shaped, highly reflective nuclei and medium-reflective cytoplasmic cells (10 *μ*m)	Same as in vivo CLSM	Monolayer, purple nuclei in the center of the pink cytoplasm, darker than stratum spinosum
Papillary dermis ↓ 60–120 *μ*m	The low-reflection area is surrounded by a highly reflective layer, and highly reflective cells (inflammatory cells, red blood cell) are visible inside	Same as in vivo CLSM	Crispy pink collagen fibers surrounded by stratum basale
Reticular dermis	Low reflection background and high reflection net-like collagen fibers	Crispy medium reflective collagen fibers	Crispy pink collagen fibers
Hair follicle	Highly reflective hair shaft surrounded by highly reflective root sheaths with cells arranged in columns	Same as in vivo CLSM	Hair shafts as columns of pink to brownish keratinized cells
Glands of Zeis	Tubular high reflection duct and blurred medium reflection acini	Round, sharply demarcated structures filled with low-reflective cytoplasm, highly reflective nuclei	Opens into the acetabulum, large cells packed with lipids and centrally located nuclei
Glands of Moll	Unable to observe	1–2 layers of small highly reflective nuclei cells, lining the low-reflective lumen	Located near the lash follicles within the margins of the lids, tubular composed of flat cells, others with cuboidal cells

↓, depth under surface.

**Table 2:** List of in vivo and ex vivo CLSM features of healthy palpebral conjunctiva part of the eyelid margin and their histological correlates in H&E staining.

Anatomical	In vivo CLSM	Ex vivo CLSM	H&E staining
Superficial epithelium ↓ 0–70 *μ*m	Tightly packed, low-reflective cell junctions and high-reflective cells with invisible nuclear (15 *μ*m), filled with small amounts of highly reflective round large cells and dendritic cells	2–5 layers, polygon, high-reflective nucleus and low-reflective cytoplasmic cells	Multiple layers of pink cytoplasm with dark granules and dark blue or purple nuclei
Basal layer of the epithelium ↓ 60 *μ*m–70 *μ*m	High-reflective cell junctions and low-reflective cells with invisible nuclear (10–15 *μ*m)	Monolayer, cube-shaped, highly reflective nuclei and medium-reflective cytoplasmic cells	Monolayer, purple nuclei in the center of the pink cytoplasm, darker than stratum spinosum
Lamina propria	Low reflection background and high reflection net-like collagen fibers	Crispy medium reflective collagen fibers	Crispy pink collagen fibers
Meibomian gland	Unable to observe	Round, sharply demarcated structures filled with low-reflective cytoplasm and highly reflective nuclei	Large cells packed with lipids and centrally located nuclei

↓, depth under surface.

## Data Availability

The data used to support this study can be made available upon request via e-mail to Dr. Wang Yujing (wyjoph@outlook.com).

## Additional Points

Research highlights: the study offers an overview of the in vivo confocal microscopy human eyelid margin characteristics in comparison to the standard histological examination and ex vivo confocal microscopy characteristics. To clarify the clinical disadvantage of the meibomian gland observation using in vivo confocal laser scanning microscopy by contrasting suspicious radiographic with tissue specimen images of the meibomian glands.
